# Does energy efficiency matter for prices of tenant-owned apartments?

**DOI:** 10.1007/s11356-022-20482-w

**Published:** 2022-05-04

**Authors:** David Stenvall, Pontus Cerin, Bo Sjö, Gazi Salah Uddin

**Affiliations:** Department of Management and Engineering, Linköping University, Linkoping, Sweden

**Keywords:** Energy performance certificates, Housing markets, Tenant-owned apartments, Energy efficiency, Green premium, D10, Q51, R20

## Abstract

In this study, we use Energy Performance Certificate (EPC) information to investigate the effect of energy efficiency on the selling price of Swedish tenant-owned apartments. While there is a large body of literature on how energy efficiency affects the sales price of single-family houses, none has exclusively focused on tenant-owned apartments. For owners of tenant-owned apartments in Sweden, heating is for a large share included in the monthly fee paid to the tenant association, which usually does not change on a short-term basis. This raises the question whether homebuyers’ incentives for acquiring energy-efficient tenant-owned apartments are large enough to be capitalized into the prices. By hedonic models and matching methods, we found mixed results. In our most optimistic scenarios, tenant-owned apartments enclosed in energy-efficient buildings are sold with a premium of approximately 0.8 to 1.2% compared to apartments in non-efficient buildings. The results in this study are not robust to all model specifications and vary across regions. In comparison with recent studies using data for single-family houses in Sweden, our detected capitalization is smaller. Our results highlight a need for targeted measures if EPCs are to be fully capitalized in prices for all dwelling types in which buyers have different economic incentives for reducing their energy consumption.

**Supplementary Information:**

The online version contains supplementary material available at 10.1007/s11356-022-20482-w.

## Introduction

Reducing the speed of climate change has in the recent decade become as a top priority for numerous governments, scientists, and institutions. The EU has set up ambitious GHG emission targets in a vision for 2050, which could pose one important step-stone for the world to reach below the Paris 2-degree target and, as the union puts it, keep it down to 1.5 °C. This will, consequently, put a lot of pressure on the housing sector to abate its considerable large share of CO_2_ emissions compared to other sectors. In this context, a common European approach to reduce the energy usage by dwellings was introduced through the Energy Performance of Buildings Directive (EPBD 2002/91/EC). The directive includes a certification scheme aimed towards dwellings, namely, the Energy Performance Certificates (EPCs).^1^ One of the main purposes of the introduction of EPCs was to decrease potential information asymmetries between owners and prospective buyers with regard to the energy performance of dwellings. Hence, these certifications are supposed to make potential buyers aware of the energy performance of their future homes or buildings (e.g., Brounen and Kok 2011; Bio Intelligence Service 2013; Cerin et al. 2014; Fuerst et al. 2015; Fuerst et al. 2016a, b; EC 2020). The increased energy transparency has also benefited other actors, e.g., mortgage lenders who can offer lower interest rates by issuing green bonds. In the case of Sweden, buildings that are newly built, sold, or rented are required to have an EPC (with a few exceptions). The Swedish EPCs contain information about the building’s energy performance, and since 2014, a grading scale that like in most other EU-member countries spans from A to G.^2^

If the financial incentives to acquire energy-efficient homes are large, this should eventually be reflected in the selling price of the dwellings. For instance, many studies discuss the idea that energy savings associated with owning energy-efficient properties may be capitalized in the price of these dwellings if the energy performance is transparent (see, e.g., Brounen and Kok 2011; Cerin 2014; Marmolejo-Duarte and Chen 2019; Aydin et al. 2019). Against this background, a vast number of studies have examined whether low energy consumption or energy-efficient EPC grades are positively reflected in the selling price of residential and commercial properties. This strand of literature emerged during the close period after the EPBD directive begun to be implemented. The existing articles related to the residential housing market often observe that energy-efficient homes, after controlling for dwelling characteristics, are sold at higher prices than less efficient homes.

A limited number of papers have explored whether energy performance impacts the selling price in Sweden. These articles have reached slightly different conclusions. Regarding studies using data from the early years of the EPC implementation, Wahlström (2016) finds no effect of energy performance on the sales price. Högberg (2013) finds that lower energy consumption increases the selling price, while Cerin et al. (2014) find price premiums for certain price and age segments of homes. Recently, Wilhelmsson (2019) used newer data and estimated the price premium of energy-efficient EPC grades to approximately 3%. However, these studies have been conducted using data for single-family houses, and not exclusively for apartments, condominiums, or other tenant-forms in multi-household dwellings. As pointed out by Fuerst et al. (2016b), apartments are in general more homogeneous compared to single-family houses and, therefore, reduce problems connected to the dwelling characteristics of the individual homes. International studies, focusing only on apartments when evaluating the capitalization effect of EPCs are, in general, rare. The few studies that exist indicate either small or insignificant price premia in the sales price for more energy efficient homes (see e.g., Fuerst et al. 2016b; Fregonara et al. (2017); Taltavull et al. 2017; Fuerst et al. 2015).

In Sweden, 42% of the population lives in multi-dwelling units such as apartments (SCB 2021). Of the households that lived in multi-dwelling units in 2020, approximately 40% lived in tenant-owned apartments (*bostadsrätter*) and nearly the entire remaining part in rental dwellings. Thus, if you own an apartment in Sweden, you typically do so indirectly by tenant ownership. This contrasts to single-family houses, in which the buyer usually receives direct ownership of the dwelling. In Sweden’s tenant-owned apartments, a tenant association is the legal owner of the properties.^3^ When a household buys an apartment, they get membership in the association. A member of the association does not actually own their apartment, but they have the right to utilize it. However, it is the member that utilizes the tenant-owned apartment that sells and earns potential profits on the apartment, not the tenant owner association.^4^ It is the associations and not the resident’s responsibility that each building that is owned by the association have a valid EPC. All apartments within the same building (or with the same EPC-ID) will have the same energy performance/grade, which is the information the buyers will observe when acquiring a tenant-owned apartment. Before the purchase, potential buyers of tenant-owned apartments have the right to see a valid EPC for the building to which the apartment belongs.

In tenant-owned apartments, heating is commonly included in the fixed monthly fee that residents are required to pay to their tenant association. While there is no exact number available, our rough approximations are that the vast majority have heating included in the fee. Thus, while energy efficiency in single-family houses is associated with lower heating costs, this is less true for tenant-owned apartments. Even though the fee can change to reflect the cost structure of the association, it does usually not do that on a short-term basis. Decisions on changed fees are almost exclusively made at the annual member meeting of the tenant association. Periods of higher energy for heating, hence, will not be reflected directly in higher energy costs for these residents. Tenants’ individual usages of energy for heating as well as water and heated water are generally not measured per apartment and will, hence, not be reflected in the individual fees. However, even if individual heating costs are not included in the monthly fee, buyers might still have some economic incentives to invest in energy-efficient tenant-owned homes. Many Swedish banks have recently started to offer “green mortgages,” which gives reductions on the mortgage interest rate if you acquire an energy-efficient home, usually graded A or B in the EPC scale. However, whether the overall incentives are large enough to be capitalized in the sales price is not yet known.

This study investigates the relationship between energy efficiency for tenant association properties and the sales prices of their enclosed tenant-owned apartments.We apply hedonic price models and use data for six Swedish cities spread over the country. Our baseline sample defines energy efficiency based on energy performance data to study how it affects tenant-owned apartment prices. For a subsample, we also investigate the effect of energy-efficient EPC grades on the sales price of the apartments.^5^ To address potential problems stemming from non-randomness in our treatment variable, we also use propensity score matching and the novel coarsened exact matching. In contrast to the majority of Swedish literature on the relationship between EPCs and housing values, we also control for neighborhood characteristics as a robustness check.

Our hypothesis that buyers of tenant-owned apartments have low incentives to buy energy efficient apartments (i.e., apartments enclosed in energy efficient buildings) is also reflected in the results. We find price premiums for energy-efficient apartments in the range of 0.8 to 1.2%. However, the results are not robust, and no significant effect is found in some of our estimations. For tenant-owned apartments enclosed in properties with EPC grades, we found weakly significant effects for the top two most energy-efficient grades (A or B) compared with less efficient grades (C–G). However, when classifying labels A–C as the energy-efficient grades, no significant effect is found. Our main contribution is that we investigate the capitalization of energy efficiency on housing prices in subsegment, tenant-owned apartments, where we believe buyers have less incentive to acquire energy-efficient homes. By focusing on this sub-sector, we add more knowledge about how energy efficiency affects apartment prices, which is a topic that has received less attention in comparison to energy efficiency and single-family house prices.

The paper is structured as follows: “Literature review” presents the previous literature, followed by “Estimation and preliminary analysis” where data and methodology are depicted. “Empirical results and discussion” outlines the results and discussion.

## Literature review

Our paper is mainly related to the studies that analyze the relationship between EPCs — primarily focusing on energy efficiency (i.e., energy performance) — and housing prices. The effect of energy efficiency on property values has been analyzed on different national housing markets with some mixed results. Following, we present the most relevant parts of this research, based on the emphasis of this paper. Even though the total number of studies is too large to make it exhaustive, more studies are summarized in Table A1 of the appendix.

**Table 1:** Summary statistics and frequency table

Panel A. Summary statistics			
Variable	Obs	Mean	Std. dev
Price	21,696	3,364,213	2,065,931
Area	21,696	64.03	24.84
Room	21,696	2.44	0.99
Age	21,696	63.98	32.84
Rent	21,696	3515.02	1344.48
Panel B. Frequency table			
Variable	Frequency	Share of full sample (%)	Share of total grades (%)
HIGH	1397	6.44	
LOW	10,287	47.41	
Grade A	39		0.59
Grade B	385		5.87
Grade C	1040		15.87
Grade D	1353		20.64
Grade E	2266		34.57
Grade F/G	1472		22.46

This table shows the descriptive stats for our control variables (panel A) and frequency table (panel B) of our variables for energy efficiency. Price is the sales price in SEK; Area is the size of the dwelling in sqm; Room is the number of rooms; Age is equal to year 2020 minus the construction year for each dwelling; Rent is the rent or fee paid to the tenant association each month in SEK; HIGH is a dummy variable for tenant-owned apartments enclosed in tenant-owned buildings with high energy efficiency; and LOW is a dummy variable for tenant-owned apartments enclosed in tenant-owned buildings with low energy efficiency. Energy grades are dummy variables equal to 1 if the tenant-owned apartment belongs to a tenant-owned building with the specified EPC grade/grades

### International evidence on the relationship between energy performance and housing prices

Brounen and Kok (2011) examine the relationship between energy labeling and housing prices in the Dutch residential housing market. The authors apply a hedonic price model with controls for neighborhood characteristics, such as housing density. By comparing the estimated selling price of homes with energy-efficient EPC grades (i.e., labels A, B, and C) to homes with the EPC label D, the authors find price premium for the former category of homes corresponding to 10, 5, and 2%. Fuerst et al. (2016a) examine how EPC ratings affect selling prices on the residential housing market in Wales. The authors find, after including a large number of control variables, that homes with labels A/B and C are sold at a higher square meter price compared to dwellings classified with labels D. For lower-efficiency labels (ratings E, F, and G), they find discounts in the sales price compared to homes with a D label. Fuerst et al. (2016a) do not observe a statistically significant price premium for A/B-rated flats in comparison to apartments with rating D. Furthermore, De Ayala et al. (2016) focus on the energy efficiency in the Spanish market. Using energy efficiency information retrieved from survey data, they examine the effect of energy efficiency on stated housing prices in Spain. By applying hedonic price models, they find that homes labeled as either A, B, C, or D have a price premium of 5.4% in comparison to homes labeled as E, F, or G. Hyland et al. (2013) focus on the Irish residential market using sales price data for apartments, terraced houses, detached houses, bungalows, and single-family houses. They find a 9.3% price premium for homes with A label compared to D level, and a 5.2% premium for label B compared to D. Recently, Jensen et al. (2016) study EPCs in Denmark and find that energy-efficient ratings have a positive impact on the residential values of single-family homes. The authors find the effect to be over 6% for the grade A/B compared with grade D. In a comprehensive study conducted by Bio Intelligence Service (2013), the authors analyze how energy performance affect sales prices of dwellings in Belgium, France, Austria, Ireland, and UK. The authors find positive relationships between energy efficiency (based on EPC information) and house prices in all investigated areas except for one subarea — Oxford, UK. Another study by Fuerst et al. (2015) explores the linkages between EPC ratings and housing prices in England. They find that dwellings labeled either A or B are sold with price premiums compared to D-labeled dwellings. Regarding the effect of energy ratings on the sales price (per sqm) for flats, their hedonic price models reveal a price premium of approximately 1.6% for label A or B compared to the hold-out category D.

Some papers investigate the effect of energy labels on housing prices in countries that use rating schemes other than the EPCs. For instance, Kahn and Kok (2014) measure the effect of green labeling in the California housing market based on the rating schemes LEED, GreenPoint, and Energy Star. The authors find a small price premium for single-family homes with a green label compared to non-labeled homes. Other studies from the USA include Bruegge et al. (2016), which focus on the “Energy Star” certifications of homes. The authors in this study find price premiums for new homes labeled with the Energy Star certification.

Other articles focus on specific cities rather than broader region- or country-level effects. Fuerst et al. (2016b) investigate how energy-efficient labels, as classified by the EPCs, affects the apartment prices in Helsinki, Finland. By applying hedonic price models, they find a price premium of 3.5% for buildings in the high-efficiency categories (A, B, and C) compared to homes with label D. However, after controlling for neighborhood characteristics, the premium decreases to 1.5%. Fregonara et al. (2017) study the impact of energy efficiency on a sample of residential apartments in Turin, Italy. By using hedonic price models, the authors find (after controlling for apartment characteristics) that EPC labels do not affect the prices. Furthermore, Taltavull et al. (2017) study the impact of energy efficiency on apartment prices in Bucharest, Romania. Their results show that energy-efficient dwellings are sold at a higher price compared to non-efficient dwellings in two out of five analyzed areas. Lastly, Marmolejo-Duarte and Chen (2019) examine whether energy efficiency affects the apartment prices in Barcelona. The authors find a premium of 7.8% for apartments with the EPC label A compared to label G. However, the authors also discover that the premium varies across different clusters in the city.

### Energy efficiency and housing prices in Sweden

There are a few studies regarding energy efficiency and housing values in a Swedish context, with mixed evidence. However, all studies have been conducted using data for single-family houses rather than tenant-owned buildings or apartments, and in most cases using data before or during the initial phase of the EPC implementation. Högberg (2013) investigate the effect of energy efficiency on housing prices in Stockholm, using a small sample from 2009. Using hedonic price models, the author shows that reductions in energy consumption of dwellings are associated with increases in the selling price. Cerin et al. (2014) conducts a larger study using data from 2009 and 2010. Their data covers sales of single-family houses taking place in cities and commuting areas during these years. Based on a sample of over 67,500 observations, they find that energy efficiency (based on EPC information) positively affects the selling price of residential houses for certain price segments and for dwellings of certain age classes. The authors also find that the effect of energy efficiency is unevenly capitalized in the selling price for different price segments and age groups. Wahlström (2016) uses a dataset with more than 75,000 observations of houses sold in Sweden during 2009 and 2010. The author finds no price premium for energy efficient dwellings. Lastly, Wilhelmsson (2019) uses a dataset consisting of residential single-family houses in Sweden based on the period 2013–2018. The author adjusts for biases in the hedonic price model by estimating the relationship between energy efficiency and housing prices with a combination of model approaches, such as robust regressions, quantile regressions, and spatial models. The results indicate that having an energy-efficient EPC grade (compared to having a non-efficient grade) increases the selling price by approximately 3%. Furthermore, Wilhelmsson (2019) finds a larger price premium in the northern part of Sweden, which belongs to a different climate zone compared with the rest of the country.

## Estimation and preliminary analysis

### Data, methodology, and estimation procedure

In this study, we analyze the relationship between the sales prices of tenant-owned apartments and the energy efficiency as stated in their confining building EPCs. The data used in this study is gathered from Svensk Mäklarstatistik AB, i.e., the association of Swedish realtors, and covers residential sales prices (based on the contract price) of tenant-owned apartments during the full period of 2019.^6^ Our data consists of observations from six municipalities that all constitute the major city in its region in Sweden: Malmö, Göteborg, Linköping, Stockholm, Luleå, and Umeå. Our motivation behind choosing these cities is to get a broad and geographically diversified picture of the relationship between housing prices of tenant-owned apartments and the energy performance of their confining buildings. In addition to the sales contract price of each individual apartment, the data includes information about the energy performance and energy performance rating of the building to which the apartment belongs to. The energy efficiency data retrieved is expressed as the tenant-owned building’s energy performance in kilowatt-hours (kWh) per square meter, while the rating system is a grade that ranges from A to G. In the grading scale, A is given to the buildings/dwellings that are most energy efficient and G to the least efficient. In Sweden, the EPCs were introduced in mid-2007 for commercial and multi-family buildings, but the grading scale was not implemented in Sweden until 2014. As the EPC is valid for 10 years, some tenant association–owned buildings only have information of their building’s energy performance, but not actual grade. Svensk Mäklarstatistik gathers the data based on reports from realtors, so the energy information we receive is based on the realtors’ reports of the energy performance and grades originating from the EPCs. In total, a quarter of our observations will have both the energy performance and grade of their confining buildings, but all observations have energy performance data. Therefore, our dataset will consist of a full sample including all apartments (with energy performance data) and a subsample only including those apartments that is enclosed in a building with a valid EPC-grade (*graded sample*). In the full sample, we use a dummy variable approach to classify our observations either as energy-efficient (i.e., high energy efficiency or low energy usage) or low-efficient apartments.

The variable for high energy efficiency (denoted as *HIGH*) is equal to one if the tenant-owned apartment belongs to a building with an energy performance equal to or below 75 kWh per square meter. The variable is equal to zero for observations enclosed in buildings with energy performance larger than 75 kWh per square meter. The low energy efficiency variable (denoted as *LOW*), which we use in a few model specifications, is equal to one if the tenant-owned apartment belongs to a building with energy performance above 140 kWh per square meter, but is otherwise equal to zero. The boundaries have been chosen to match the energy grades. Approximately 6.5% of the sample has an energy performance below 75 kWh, which is similar to the share in the graded sample with either A or B as EPC rating (see Table 1).^7^ In the graded sample, we create two alternative variables for energy efficient apartments, that are used in different model specifications. First, the variable *AB* is dummy variable equal to one for tenant-owned apartments enclosed in buildings with energy rating A or B. This variable is equal to zero for apartments enclosed in buildings with other ratings. Similarly, the variable *ABC* is a dummy variable equal to one for tenant-owned apartments enclosed in buildings with energy grade A, B or C, but equal to zero for apartments enclosed in buildings with other energy grades.

The data also includes control variables for each observation (i.e., tenant-owned apartment), such as the number of rooms, building year, area in square meters, and the rent or fee paid to the tenant-association each month. In the retrieved data, a small share of the observations has two different construction years, one older and one much younger, assumingly because the apartments have been significantly renovated or rebuilt since the building year. We let the oldest of the 2 years represent the age of the building, but add a dummy variable equal to 1 for these observations to control for potential effects of renovations. The dataset has been cleaned from missing data and other outliers. A few apartments in our sample (out of the nearly 22,000 observations) were more than 300 years old and clearly differed from the rest of the sample. We therefore excluded these. We also excluded some observations that had extreme values for the energy performance. Given the large dataset and the design of our estimations (with dummies for age and energy performance), we believe that this is a minor issue.

To achieve the aim with our study, we start by estimating hedonic price models, a method that was introduced in Rosen (1974). The hedonic price models are common when it comes to explaining variation in housing prices and have been used in most studies regarding EPC and housing values (see e.g., Cerin et al. 2014; Wahlström, 2016; De Ayala et al. 2016; Fuerst et al. 2016a, b). We set up our baseline hedonic price model in accordance with Eq. (1):

1 $$\mathrm{ln}\left({y}_{i}\right)= \alpha + {\beta }_{1}{EE}_{i}+ {{\beta }_{2}D}_{i}+ {\beta }_{3}{\mathrm{Z}}_{i}+ {\beta }_{4}{T}_{i}+{\varepsilon }_{i}$$

Note that $\mathrm{ln}\left(y\right)$ is the natural logarithm of the sales price for each observation $i$, $\alpha$ is a constant, and ${\varepsilon }_{i}$ is the error term. Denoted as ${EE}_{i}$, it is the vector of dummy variables for energy-efficient tenant-owned apartments. These are dummy variables equal to 1 if unit $i$ is enclosed in an energy-efficient building, but equal to zero if unit $i$ is not located in an energy-efficient tenant-owned building. When using the full sample with energy performance data, ${EE}_{i}$ corresponds to the dummy variable *HIGH*. In the subsample with energy ratings, ${EE}_{i}$ corresponds to either variable *AB* or *ABC.* Note that ${D}_{i}$ is a vector of dwelling-specific attributes for observation $i$. This vector will in all model specifications include the natural logarithm of the area, the number of rooms, the natural logarithm of the fee paid to the tenant association, and dummy variables for the building year of the apartments. To control for locational effects, we include postal code dummy variables. Hence, this means that we create dummy variables equal to 1 if observation $i$ belongs to a certain postal code, and else equal to zero. We only use the first four digits in the postal code if nothing else was stated. To avoid perfect multicollinearity, we create $K-1$ locational dummy variables where $K$ is the number of postal codes (based on the first four digits). While the Swedish postal codes are five-digit numbers, the inclusion of dummy variables based on the full number of digits would lead to very few observations per postcode, as the five digits represent a very small geographical area. The first four digits represent a much smaller area than municipality fixed effects (hence, we control more in detail for locational effects), but we get more observations per postcode compared to using the five digits. The postal code fixed effects are represented by ${Z}_{i}$ in Eq. (1), and the vector of coefficients by ${\beta }_{3}$. Lastly, we create 11 dummy variables to control for the contract month of the apartment sales (January omitted). Hence, ${T}_{i}$ is the vector for these time fixed effects. Our hedonic price models consist of cross-sectional regressions estimated with OLS.

As pointed out by Wilhelmsson (2019), the hedonic price model can be sensitive to certain biases such as spatial dependence, omitted variables, and outliers. While the spatial dependence is controlled for by postal code dummy variables, we also include neighborhood control variables as a robustness check in Tables 5 and 6. The included variables are median net income (in thousands of SEK), share of persons with foreign origin, share of persons in rental apartments, share of persons receiving financial aid, and number of households. In the estimations, we express the net income and number of households in logarithmic form. All these variables are gathered from the database DeSO from Statistics Sweden. The DeSO regions are small statistical areas different from our postcodes. Hence, even if we only control for the 4 digits in the postcode, we can include the neighborhood controls to explain the data on an even more detailed level. Because DeSO areas are geocoded, each observation, i.e., sold apartment, is matched to a specific DeSO based on their coordinate. To mitigate issues related to outliers or influential outliers, we also run the hedonic estimation with robust regression (rreg in Stata) along with our OLS estimations. By robust regression, observations with a Cook’s distance larger than 1 are omitted and observations with a large absolute residual are given less weights in the final estimation procedure (see e.g., Fuerst et al. 2020 for studies using robust regression when analyzing capitalization in energy-efficient dwellings).

Some authors have pointed out that classic hedonic price estimations might suffer from sample selection bias (see, e.g., Marmolejo-Duarte and Chen 2019; Wilhelmsson 2019) or other methodological drawbacks (Aydin et al. 2020). In our case, apartments that are energy efficient (treatment group) might have different characteristics from those that are not energy efficient (control group). Thus, the treatment effect might be distributed non-randomly. For instance, it is more likely that older buildings have lower energy efficiency compared to newer ones. Hence, we will have a discrepancy between treated and control groups regarding the age of the buildings. Even though we control for the age of the apartments, we cannot be certain that this relationship is modeled completely correct regarding aspects as the functional form of this relationship, such as linear or non-linear (see for instance a discussion on this issue in Black and Smith 2004). To reduce such problems, it is possible to use some matching methods. By matching, we try to compare energy-efficient dwellings with non-efficient dwellings that are more alike regarding other important factors, such as age, level of rent, numbers of rooms, and location. Thus, we aim to make the treatment and control groups more similar to each other. In this study, we will use two different types of matching, the propensity score matching (PS) and the coarsened exact matching (CEM). The propensity score by Rosenbaum and Rubin (1983) is obtained by running a binary regression with the treatment, in our case apartments that are energy efficient, as the dependent variable and other covariates as independent variables. The treatment and control groups are then matched based on their estimated probabilities from the binary regression. Propensity scores have been used in earlier studies related to capitalization of energy efficiency in dwellings (see, e.g., Eichholtz et al. 2013; Walls et al. 2017; Wilhelmsson 2019). We also implement the CEM by Iacus et al. (2012) as an alternative method to address the possible confounding. CEM works by coarsening variables to groups, where the members in a certain group are given the same numerical value. Identical observations that have identical values in all coarsened groups are then matched together into a stratum and given a weight, with matched treatments assigned a weight of 1.^8^ Stratums without at least one control and treatment observation will be omitted, and a weight of 0 is assigned to unmatched units and is, thus, omitted (for more details, see, e.g., Iacus et al. 2012 and Blackwell et al. 2009). The weights obtained from the CEM can then be used to weight the hedonic price estimations. To the best of our knowledge, we do not find any authors that have used CEM to investigate the capitalization of energy efficiency in housing prices. It has, however, been applied in related fields — i.e., public green procurement (e.g., Simcoe and Toffel 2014).

In our estimations, we start by estimating the hedonic price model in the full sample. We also run subsamples to control if the effect on prices of tenant-owned apartments enclosed in energy efficient tenant-owned buildings is different between regions. Furthermore, we run subsamples for those observations enclosed in buildings with an energy grade (graded sample). To get a full picture of how energy efficiency affects the prices in the tenant-owned apartments sector, we test different specifications of the base value to which we compare the high- and low-efficiency grades. We thereafter proceed by running the hedonic estimation while controlling for neighborhood characteristics. Lastly, we use propensity score and coarsened exact matching to mitigate potential issues related to confounding.

### Descriptive statistics

Table 1 shows the descriptive statistics and a frequency table of our included sample. As shown, our typical object is a tenant-owned home with 2.4 rooms with an average area of 64 m^2^. The average price of over 3.3 million SEK is quite high compared to the average tenant-owned home in Sweden. This is caused by the fact that a large part of our sample consists of objects in the municipality of Stockholm and Gothenburg that has a higher price level compared to the rest of the country. As further observed, only a small share of the apartments is classified as energy efficient (HIGH) according to our definition. In the sample with energy ratings *(graded sample)*, we observe that over 6% of the sample has energy rating A or B.

## Empirical results and discussion

### Hedonic price estimations

Table 2 shows the results from the hedonic price estimations for the full sample. In this sample, we use dummy variables for tenant-owned apartments enclosed in energy-efficient tenant-owned buildings, as measured by the energy performance of the building’s EPC. Such apartments will hereafter be denoted as *energy-efficient apartments.*

**Table 2:** Full sample — hedonic price estimations

	(1)	(2)	(3)	(4)	(5)
*Energy efficiency (EPC)*					
HIGH	0.001 (0.007)	0.011** (0.005)	0.011** (0.005)	0.007* (0.004)	0.007* (0.004)
LOW			− 0.001 (0.002)		− 0.001 (0.002)
*Dwelling-specific controls*					
Area	0.862*** (0.019)	0.746*** (0.012)	0.746*** (0.012)	0.751*** (0.006)	0.751*** (0.006)
Rent	− 0.277*** (0.023)	− 0.144*** (0.014)	− 0.143*** (0.014)	− 0.156*** (0.004)	− 0.156*** (0.004)
Room	0.049*** (0.004)	0.060*** (0.002)	0.060*** (0.002)	0.058*** (0.002)	0.058*** (0.002)
Building year					
1901–1921	− 0.041***	− 0.002	− 0.002	− 0.006	− 0.006
1922–1941	− 0.143***	− 0.066***	− 0.066***	− 0.070***	− 0.070***
1942–1961	− 0.280***	− 0.116***	− 0.116***	− 0.120***	− 0.120***
1962–1976	− 0.467***	− 0.165***	− 0.165***	− 0.163***	− 0.163***
1977–1991	− 0.304***	− 0.143***	− 0.144***	− 0.139***	− 0.139***
1992–2005	− 0.218***	− 0.074***	− 0.074***	− 0.063***	− 0.063***
2006–2019	− 0.123***	− 0.001	− 0.002	− 0.004	− 0.004
Constant	13.48***	13.48***	13.48***	13.54***	13.54***
Time fixed effects	Yes	Yes	Yes	Yes	Yes
Robust regression	No	No	No	Yes	Yes
Postal code fixed effects	(2 digits)	Yes	Yes	Yes	Yes
*R*^2^	0.812	0.933	0.933	0.943	0.943
Obs	21,696	21,696	21,696	21,695	21,696

The dependent variable is the logarithmic sales price. Standard errors in parenthesis. Columns (1), (2), and (3) are estimated with robust standard errors. Postcode fixed effects is based on 4 digits if nothing else is stated. HIGH is a dummy variable for tenant-owned apartments enclosed in tenant-owned buildings with high energy efficiency; LOW is a dummy for tenant-owned apartments enclosed in tenant-owned buildings with low energy efficiency; Area is the size of the dwelling in square meters (log); Room is the number of rooms; Building year (age) is a dummy equal to 1 if the apartment was constructed during the given range of years (omitted category is pre-1901). Rent is the fee paid to the tenant association each month in SEK (log). We also include a dummy variable equal to 1 for apartments with double construction years (see the explanation in the data section), for which the result is not shown

Statistical significance is denoted by ***p < 0.01, **p < 0.05, *p < 0.1

In Table 2, column (1), we start by controlling for a small geographical area by the first two digits in the postcode. We observe that all our control variables are significant with the most variation in sales price explained by the size of the home (area) and the rent level (monthly fee paid to association). The negative coefficient of the rent level is expected as higher rent increases the monthly costs for homeowners. The significant dummy variables for the age of the apartments indicate a significant difference in price compared to tenant-owned apartments constructed before 1901. The negative sign is not unexpected as the reference category is expensive apartments mostly located in concentrated regions in the city center of Stockholm. Further, we find that the area of the tenant-owned homes in our sample is correlated with the number of rooms according to the VIF values (variance inflation factors). As both are control variables, they are kept in the estimations. The VIF value for our treatment variable for energy-efficient tenant-owned apartments, HIGH, is very low and does not suggest any problems with multicollinearity. Our *R*^2^ over 81% is notably high, but common for hedonic price studies on the housing market, especially given that our sample consists of apartments which are more homogeneous compared to houses. As HIGH is insignificant, we do not find any effect of energy efficiency on the sales price of the tenant-owned apartments.

In column (2), we instead use the 4-digit postcode to control for more detailed locational effects. Our *R*^2^ now increases to 93%, which indicates that a more detailed control for location is important in explaining the sales price. Our treatment, HIGH, is now significant and indicates a price premium of approximately 1.1% for energy-efficient tenant-owned apartments. This can be interpreted as approximately 11,000 SEK more for every 1 million in sales price for the energy-efficient homes, ceteris paribus, compared to the reference category (homes enclosed in buildings with higher energy performance than 75 kWh/m^2^). The size of the premium is smaller compared with the findings of Cerin et al. (2014) and Wilhelmsson (2019) that found larger premiums when using data for houses rather than apartments. As emphasized in “Introduction,” a large share of the homeowners of tenant-owned apartments has heating included in the monthly fee paid to the association each month, which usually does not change on a short-term basis. The fee is not based on the usages per apartment but rather the joint costs for the association. That the premium is quite low might simply reflect low economic incentives for many buyers of tenant-owned apartments. In column (3), we include our dummy, LOW, for tenant-owned apartments enclosed in tenant-owned buildings with low energy efficiency (hereafter denoted as *low-efficient apartments*). By including dummies for apartments with both high and low energy efficiency, the reference category is now apartments in between these two categories (medium energy efficient). The insignificance of LOW indicates that low-efficiency apartments are not sold with a discount compared to medium-efficiency homes all else equal. In columns (4) and (5), we try to mitigate issues related to potential outliers by performing the estimations using the robust regression. The effect of high energy efficiency on the sales price is now reduced to a premium of approximately 0.7% that now is weakly significant. The effect of low efficiency on prices is still insignificant.

In Table 3, we present the result from regional subsamples. The category South consists of the observations from the municipality of Malmö while the Mid category is based on the sales from Gothenburg and Linköping. As only a few observations of the sample from the northern cities Luleå and Umeå do qualify as energy efficient according to our boundaries, we do not run estimation solely based on these observations. As observed, the coefficient for HIGH is largest in the southern sample followed by the Stockholm. The effect is, however, not statistically significant for any region.

**Table 3:** Regression with regional subsamples from the full sample

	Stockholm	South	Mid
HIGH	0.004 (0.006)	0.015 (0.017)	0.002 (0.008)
Dwelling-specific controls	Yes	Yes	Yes
Time fixed effects	Yes	Yes	Yes
Postal code fixed effects	Yes	Yes	Yes
*R*^2^	0.913	0.885	0.912
Obs	11 468	4142	5285

The dependent variable is the logarithmic sales price. Standard errors are in parenthesis. Postcode fixed effects are based on 4 digits. Robust standard errors are used in the estimations. The mid region contains dwellings from the municipalities Linköping and Gothenburg, and the southern region consists of apartments from the Malmö municipality. HIGH is a dummy for tenant-owned apartments enclosed in tenant-owned buildings with high energy efficiency. Dwelling-specific variables include the following: area (log), number of rooms in each apartment, rent or fee paid to the tenant association each month (log), and dummy for construction year (same as Tables 2 and 3). We also include a dummy variable equal to 1 for apartments with double construction years (see the explanation in the data section)

Statistical significance is denoted by ***p < 0.01, ** *p* < 0.05, * *p* < 0.1

Table 4 shows the hedonic price estimations using the sample of tenant-owned apartments enclosed in a tenant-owned building with an EPC grade. In (1), we classify energy-efficient apartments as those enclosed in a building with the grades A or B and the other labels (C–G) as the reference category. Our results indicate that the effect of energy efficiency is positively related to the sales price but only weakly significant. The estimated premium is approximately 1.5%, all else equal, which is higher compared with the effect of energy efficiency in the full sample in Table 2. This 1.5% premium can be compared with the ten-basis point reduction in mortgage rate that buyers can get by certain banks if they acquire a home with an energy grade of A or B. Assuming an annual mortgage rate of 2% on a 4 million SEK loan, a 0.10-percentage point reduction is approximately 4000 SEK less per year. If not considering the time value of money or any other potential cost savings from owning an energy-efficient home, a 1.5% premium is paid off in 15 years based solely on the mortgage rate reduction. Therefore, a 1.5% premium is not unreasonable based on these economic incentives. In Table 4, column (2), we instead define the energy-efficient labels as A–C and compare it with the less efficient grades (D–G). Even though the coefficient for the energy efficient grades is positive, the effect is not significant. Hence, our results from (1) and (2) suggest that the price premium is only present in the most energy-efficient labels, A and B. That these two labels also provide the largest interest rate reduction for homebuyers with green loans might perhaps be a part of the explanation. In column (3), we add dummies for the least energy efficient labels (E–G). Our result from this estimation shows no significant discount compared with the reference category (label D). In (4) and (5), we run the estimations using robust regression. Our effect found in column (4) indicates a 1% premium for labels A and B. However, this is not enough to reach statistical significance. While the robust regression barely drops any observations, it still weights the observations that are not dropped. Hence, the reduced effect from 1.5% premium in column (1) to 1% in column (4) is likely due to the weighting of potential outliers rather than the actual dropping of observations.

**Table 4:** Grade sample — hedonic price estimations

	(1)	(2)	(3)	(4)	(5)
*Energy grade (EPC)*					
AB	0.015* (0.009)			0.010 (0.008)	
ABC		0.001 (0.005)	0.002 (0.006)		− 0.001 (0.006)
EFG			0.002 (0.005)		− 0.002 (0.005)
*Dwelling-specific controls*					
Area	0.749*** (0.023)	0.750*** (0.023)	0.750*** (0.023)	0.750*** (0.012)	0.750*** (0.012)
Rent	− 0.164*** (0.025)	− 0.165*** (0.025)	− 0.165*** (0.025)	− 0.170*** (0.009)	− 0.170*** (0.009)
Room	0.065*** (0.004)	0.065*** (0.004)	0.065*** (0.004)	0.059*** (0.003)	0.059*** (0.003)
Building year					
1901–1921	0.000	0.000	0.000	0.000	− 0.001
1922–1941	− 0.059***	− 0.059***	− 0.060***	− 0.068***	− 0.068***
1942–1961	− 0.129***	− 0.129***	− 0.129***	− 0.136***	− 0.136***
1962–1976	− 0.172***	− 0.172***	− 0.172***	− 0.165***	− 0.165***
1977–1991	− 0.166***	− 0.166***	− 0.166***	− 0.158***	− 0.158***
1992–2005	− 0.091***	− 0.091***	− 0.091***	− 0.079***	− 0.079***
2006–2019	0.016	0.019	0.019	0.013	0.014
Constant	13.80***	13.80***	13.80***	13.97***	13.97***
Time fixed effects	Yes	Yes	Yes	Yes	Yes
Robust regression	No	No	No	Yes	Yes
Postal code fixed effects	Yes	Yes	Yes	Yes	Yes
*R*^*2*^	0.936	0.936	0.936	0.945	0.945
Obs	6555	6555	6555	6555	6553

The dependent variable is the logarithmic tenant-owned apartment sales price. Standard errors in parenthesis. Columns (1), (2), and (3) are estimated with robust standard errors. Postcode fixed effects is based on 4 digits if nothing else is stated. Area is the of the size of the dwelling in sqm (log); Room is the number of rooms; Building year (age) is a dummy equal 1 if the apartment was constructed during the given range of years (omitted category is pre 1901). Rent is the fee paid to the tenant association each month in SEK (log); Energy grades are dummy variables equal to 1 if the tenant-owned apartment belongs to a tenant-owned building with the specified EPC-grade/grades. In columns (3) and (5), the energy grade D is the omitted category, while grades C–G is omitted in columns (1) and (4) and D–G in (2). We also include a dummy variable equal to 1 for apartments with double construction years (see explanation in the data section), for which the result is not shown. Columns (1), (2), and (3) are ordinary hedonic price estimations while (4) and (5) are hedonic price model estimated by robust regressions

We denote statistical significance by ***p < 0.01, **p < 0.05, *p < 0.1

### Robustness check: neighborhood controls and matching

As one of the few Swedish studies, we also control for some neighborhood characteristics in our hedonic price models. In this section, we use the full sample from Table 2. Because the neighborhood data is based on DeSO regions, observations belonging to the same region will get the same neighborhood values. In Table 5, columns (1) and (2) we start the investigation by dividing the data into income categories based on the income data. We calculate the income quartiles in the sample based on the incomes attached to each observation. Apartments with incomes below quartile 1 is denoted by IC1, apartments with incomes equal to or larger than quartile 1 but smaller than quartile 2 is denoted by IC2, and, lastly, apartments with incomes equal to or larger than quartile 2 but smaller than quartile 3 is denoted by IC3.

**Table 5:** Full sample — hedonic prices with neighborhood controls

	(1)	(2)	(3)	(4)	(5)
HIGH	0.008* (0.005)	0.006 (0.004)	− 0.003(0.005)	0.003 (0.005)	0.000 (0.004)
IC1	− 0.113***	− 0.092***			
IC2	− 0.053***	− 0.038***			
IC3	− 0.022***	− 0.015***			
Dwelling-specific controls	Yes	Yes	Yes	Yes	Yes
Time fixed effects	Yes	Yes	Yes	Yes	Yes
Robust regression	No	Yes	No	No	Yes
Postal code fixed effects	(4 digits)	(4 digits)	(3 digits)	(4 digits)	(4 digits)
Neighborhood controls	No	No	Yes	Yes	Yes
*R*^2^	0.935	0.944	0.924	0.938	0.946
Obs	21,696	21,691	21,696	21,696	21,696

The dependent variable is the logarithmic tenant-owned apartment sales price. Standard errors in parenthesis. HIGH is a dummy variable for tenant-owned apartments enclosed in tenant-owned buildings with high energy efficiency. IC1 is a dummy for observations that belong to a postcode with low incomes. IC2 is a dummy for low to median income postcodes while IC3 is a dummy for median to upper incomes. Neighborhood controls consists of median net income (log), share of persons with foreign origin, share of persons in rental apartments, and share of persons receiving financial aid and number of households (log). Dwelling-specific variables are area (log), number of rooms in each apartment, rent or fee paid to the tenant-association each month (log), and dummy for construction year (same as Tables 2 and 4). We also include a dummy variable equal to 1 for apartments with double construction years (see explanation in the data section)

We denote statistical significance by ***p < 0.01, **p < 0.05, *p < 0.1

As observed in Table 5, column (1), the income dummies (IC 1–3) are all significant with negative coefficients. This means that there is a significant difference in price, all else equal, between observations within our included income categories and our omitted reference group (high incomes above the third quartile). Our energy efficiency variable, HIGH, indicates a price premium of approximately 0.8%, but it is only of a weakly statistically significant size. However, when using 3-digit postcodes (a wider geographical area) in column (3), controlling for outliers using robust regression in (2) and (5), including a full set of neighborhood controls (3)–(5), our energy efficiency variable HIGH is no longer significant.

In Table 6, we test if our estimations vary depending on the boundaries for which we classify the apartments as energy efficient. As observed, the coefficient when narrowing the boundaries, columns in (1) estimate of HIGH, is larger compared to the results in Table 5, but still not significant. This might be a sample size problem with relatively few observations reaching energy efficiency status. If instead increasing the boundaries in (2), we observe a non-significant premium for energy-efficient tenant-owned apartments of approximately 0.5%. In (3), we include an interaction between our energy efficiency measure and the net income in the DeSO in which each observation belongs to. By this, we want to analyze if the effect of energy efficiency on prices is different if the energy-efficient apartments are sold in a low- versus high-income area. As a comparison, an energy-efficient apartment in a neighborhood with a net income of 250,000 SEK (which is slightly below median in the sample) will according to our estimates be sold with a premium of 1.77% compared to a non-efficient apartment. However, in a neighborhood with a net income of 320,000 SEK (above median), an energy-efficient apartment is sold with a discount of − 0.2%. The interaction between net income and HIGH is significant even if we run the estimation with our standard boundary of 75 kWh/sqm. One possible explanation is that there might be a larger share of apartments in which heating is not included in the monthly fee in low- versus high-income areas. In such case, the economic incentives might be lower for high-income individuals. It could also be that high-income individuals simply do afford to not care about energy savings. As we do not have information on heating included for the full sample, we are not able to control for this.

**Table 6:** Robustness and alternative boundaries for energy efficiency

	(1)	(2)	(3)
HIGH (≤ 50 kWh/m^2^)	0.010 (0.012)		
HIGH (≤ 100 kWh/m^2^)		0.005 (0.003)	0.468*** (0.098)
HIGH (≤ 100 kWh/m^2^) × net income			− 0.082*** (0.017)
Neighborhood controls	Yes	Yes	Yes
Dwelling-specific controls	Yes	Yes	Yes
Robust regression	No	No	No
Time fixed effects	Yes	Yes	Yes
Postal code fixed effects	(4 digits)	(4 digits)	(4 digits)
*R*^2^	0.938	0.938	0.938
Obs	21,696	21,696	21,696

The dependent variable is the logarithmic tenant-owned apartment sales price. Standard errors are in parenthesis. HIGH is a dummy for tenant-owned apartments enclosed in tenant-owned buildings with high energy efficiency. Dwelling-specific variables consist of the following: area (log), number of rooms in each apartment, rent or fee paid to the tenant-association each month (log), and dummy for construction year (same as Tables 2 and 4). Neighborhood controls include median net income (log), share of persons with foreign origin, share of persons in rental apartments, share of persons receiving financial aid, and number of households (log). We also include a dummy variable equal to 1 for apartments with double construction years (see explanation in the data section)

Statistical significance is denoted by ***p < 0.01, **p < 0.05, *p < 0.1

As described in the data section, ordinary hedonic price estimations might, however, suffer from methodical problems. Using a propensity score method, we can address the issue of differences between the group with high energy efficiency (treatment) versus the non-energy efficient group (control). In Table 8, we use two different methods of propensity score. First, we estimate the propensity score using a probit model and include the score in the hedonic model as a covariate. The propensity score in the first step is estimated using the treatment variables (HIGH and AB) as the dependent variable and the dwelling- and location-specific variables from the hedonic estimations as independent variables. Here, we chose to only include dummies with high energy efficiency and not for low efficiency. In the second method, we match the treatment and control groups based on the estimated propensity score using a nearest neighbor approach. We thereafter run the hedonic regression on this matched sample using weights from the matching process. For a discussion of these methods, see, e.g., Stuart (2010).

Table 7 shows the means in the sales price and covariates before and after propensity score matching. As suspected, we can observe that the treatment group in the unmatched samples largely differs regarding the age of the tenant-owned apartments. The control groups include older apartments that are both smaller and cheaper on average. However, after the matching procedure these differences have been largely reduced. The balance in the matched samples is tested by *t*-tests and mean standardized differences, which can be found in appendix Table A3. These tests show that there is no significant difference between the control and treatment groups after matching. Table 8 shows the results from the propensity score estimations.

**Table 7:** Mean in covariates before and after propensity score matching

	Full sample				Graded sample			
	*Unmatched*		*Matched*		*Unmatched*		*Matched*	
	*Treatment*	*Control*	*Treatment*	*Control*	*Treatment*	*Control*	*Treatment*	*Control*
Price	3,742,086	3,338,207	3,761,089	3,754,787	3,605,326	3,129,489	3,697,648	3,624,645
Area	70.9	63.6	71.1	72.5	70.2	65.9	70.1	69.0
Age	26.5	66.6	26.8	28.8	19.27	53.6	20.0	23.0
Rent	3800.4	3495.4	3805.1	3975.4	3841.6	3637.1	3839.4	3814.1
Room	2.7	2.4	2.7	2.8	2.7	2.5	2.7	2.6
Obs	1397	20,299	1377	1377	424	6131	394	394

This table shows the treatment and control groups before and after propensity score matching. The treatment in the full sample is the variable HIGH, which is a dummy for tenant-owned apartments enclosed in tenant-owned buildings with high energy efficiency. The treatment for the graded sample is dummy variables equal to 1 if the tenant-owned apartment belongs to a tenant-owned building with the energy grade A or B. The matched full sample is equivalent to the sample used in estimations of column (2) in Table 8. For the graded matched sample, this is equivalent to the data used in estimations of column (4) in Table 8

**Table 8:** Result after propensity score estimations

	Full sample		Graded sample	
	(1)	(2)	(3)	(4)
HIGH	0.009* (0.005)	0.012** (0.005)		
AB			0.011 (0.009)	0.014 (0.009)
Dwelling-specific controls	Yes	Yes	Yes	Yes
Time fixed effects	Yes	Yes	Yes	Yes
Postal code fixed effects	Yes	Yes	Yes	Yes
Regression on matched sample	No	Yes	No	Yes
Propensity score as control	Yes	No	Yes	No
*R*^2^	0.927	0.911	0.929	0.939
Obs	16,511	2754	3638	788

This table shows the hedonic price model with estimated propensity score as a covariate columns (1) and (3) as well as on propensity score matched samples (columns (2) and (4)). The dependent variable is the logarithmic tenant-owned apartment sales price. Standard errors are in parenthesis. HIGH is a dummy for tenant-owned apartments enclosed in tenant-owned buildings with high energy efficiency. Postcode fixed effects are 4 digits if nothing else is stated. The treatment for the graded sample is dummy variables equal to 1 if the tenant-owned apartment belongs to a tenant-owned building with the energy grade A or B. Dwelling-specific variables include area (log), number of rooms in each apartment, rent or fee paid to the tenant-association each month (log), and dummy for construction year (same as Tables 2 and 3). We also include a dummy variable equal to 1 for apartments with double construction years (see explanation in the data section)

Statistical significance is denoted by ***p < 0.01, **p < 0.05, *p < 0.1

As we can see from columns (1) and (3), including the propensity score as a covariate largely reduces the effect of high energy efficiency on the sales price of tenant-owned apartments. The coefficient is positive and ranges from a 0.9 to 1.1% premium, ceteris paribus, for tenant-owned apartments enclosed in energy-efficient tenant-owned buildings compared to non-efficient. While the effect in the full sample is weakly significant, the estimated premium in the graded sample is insignificant. Looking at the matched samples (columns (2) and (4)), the effect is similar with the coefficient ranging from 1.2 to 1.4% price premium. As before, the estimations based on the full sample is weakly significant but the effect in the graded sample is insignificant. Regarding the insignificance in column (4), it is possible that the lack of significance could be a power issue considering the few observations. The coefficient for the treatment variable (*AB*) does not change much compared to the hedonic price model estimates in Table 4, column (1). We also observe that the coefficient for energy efficiency in Table 8, column (2), i.e., the non-graded sample, decreases compared to its corresponding hedonic estimates in Table 2. In this sample, we do not have the same issue with power, which indicates that other problems (such as non-randomness or sample selection) potentially may explain the differences between Tables 2 and 8.

Lastly, we use an alternative matching method, the coarsened exact matching (CEM) by Iacus et al. (2012). The results of the CEM are dependent on the variables chosen for the matching procedure and the cut points through which these variables are coarsened. We chose to match on the most important control variables age, log rent, log area, and the number of rooms in each tenant-owned apartment. While running the CEM in Stata, it provides an algorithm to automatically choose the cut points. However, we chose the cut points by our own to ensure that this is done based on theoretical knowledge.^9^ We test different specifications before we end up with the final cut points. To quantify how much our cut points reduce the imbalances between the control and treatment observations, we use the multivariate L1 distance. The goal is to produce a model with as low a distance as possible. The distance in our chosen models can be found in Table A4, which are lower compared with the multivariate L1 distance produced by the Stata algorithm. In accordance with the procedure of Blackwell et al. (2009), we add the matched control variables to the hedonic regression to control for the remaining imbalances. We also add the control variables that were not used in the matching. Our results are shown in Table 9.

**Table 9:** Hedonic estimations after coarsened exact matching (CEM)

	Full sample	Graded sample
HIGH	0.005 (0.005)	
AB		0.007 (0.009)
Dwelling-specific controls	Yes	Yes
Time fixed effects	Yes	Yes
Postal code fixed effects	Yes	Yes
*R*^2^	0.931	0.933
Obs	17,702	4373

This table shows the results of hedonic price estimations weighted on the matched dataset after CEM. Standard errors are in parenthesis. The dependent variable is the logarithmic tenant-owned apartment sales price. HIGH is a dummy for tenant-owned apartments enclosed in tenant-owned buildings with high energy efficiency. Postcode fixed effects are based on 4 digits if nothing else is stated. The treatment for the graded sample is dummy variables equal to 1 if the tenant-owned apartment belongs to a tenant-owned building with the energy grade A or B. Dwelling-specific variables include area (log), number of rooms in each apartment, rent or fee paid to the tenant-association each month (log), and dummy for construction year (same as Tables 2 and 3). We also include a dummy variable equal to 1 for apartments with double construction years (see explanation in the data section)

As observed, the results from the CEM are lower compared to the estimates produced from the propensity score or hedonic estimation in Tables 2 and 4. The estimated effect for the graded or full sample is not statistically significant. The overall result from the matching procedures gives some conflicting evidence. Although the propensity score approach in Table 7 indicates price premiums of energy efficiency close to the hedonic price model in Tables 2 and 4, the premiums indicated by the CEM are much lower. While results from the CEM are sensitive to the chosen cut points, the propensity score estimates are sensitive to which control observations the treatments are matched to. It is therefore very difficult to conclude which of these results are the most credible. In the previous literature, Wilhelmsson (2019) found that the price premium for single-family houses was substantially reduced when controlling for selection bias using propensity score matching. Against this background, we are not able to rule out that the results from the hedonic price models in Tables 2 and 4, at least to some extent, could be affected by sample selection bias. If such bias exists, our results from this section show that the price premium for energy-efficient tenant-owned apartments is lower compared with the results presented in Tables 2 and 4.

## Conclusion and policy implications

A plentiful of research has been conducted trying to evaluate the effect of EPCs on the price of properties. The literature has, however, in many cases such as in Sweden, focused on single-family houses rather than apartments or tenant-owned homes in multi-family buildings—this despite that both single-family houses and tenant-owned buildings are required to have a valid EPC. A large share of buyers of tenant-owned apartments has heating included in the fixed monthly fee that they required to pay to their tenant association. Hence, our hypothesis is that the incentives of paying more for an energy-efficient tenant-owned apartment is lower compared to buyers of single-family houses. Therefore, we test this hypothesis by analyzing the effect on the sale prices for tenant-owned apartments from being enclosed in an energy-efficient tenant-owned building. To do so, we performed hedonic price estimations and different matching methods with various model specifications. Our results indicate that there is a premium for tenant-owned apartments enclosed in buildings that are most energy efficient or has the highest EPC grades (A or B). The size of the premium spans from approximately 0.8 to 1.2% depending on the estimated model. However, our results vary depending on the included controls, sample, or method, and no significant premium is found in many of the estimated models. In comparison with recent studies using data for single-family houses in Sweden, our results indicate a lower premium.

Even though this study only concerns a special segment of the Swedish housing market, our findings can have important implications for future policy in other countries with the design of their energy programs. If the goal of the EPCs is to increase the incentives for buyers to pay more for energy-efficient housing, legislators need to be aware that the effect of EPCs or other energy programs might be heterogeneous across different types of buyers, and for different types of housing contracts. Hence, a one-fits-all policy is not necessarily appropriate for the EPCs to be integrated into the prices of all types of housing segments, given that the financial incentives for investing in energy-efficient homes might differ. Based on our results, we argue that more actions may be needed to address certain groups. This could also be a great opportunity for future research to investigate if this relationship holds for other housing segments.

## Supplementary Information

Below is the link to the electronic supplementary material.

Supplementary file1 (DOCX 29 KB)

## Data Availability

The authors do not have the right to share the data. Code is available on request.

## Footnotes

1 For more information regarding the EPC in EU, see e.g., EC (2020). In the Swedish case, more information can be found on the website of the National Board of Housing, Building and Planning (Boverket 2019).

2 The EPCs in Sweden are set up by independent certified experts (Wilhelmsson 2019; EC 2020). These experts measure the building’s energy performance and decide on energy classification. A is the grade for the most energy-efficient buildings while the label G is given to the least efficient buildings.

3 Apartments are the most common type of housing for tenant-owned homes. Only a small fraction of tenant-owned homes consists of terraced houses or other living spaces. Hence, from the following, we will denote the tenant-owned homes as tenant-owned apartments even though our data also includes a small share of other type of living spaces that are tenant-owned.

4 See Wimark et al. (2020) for a detailed description of different tenure types in Sweden.

5 As later described in “Estimation and preliminary analysis,” prior to 2014, Sweden applied an energy usage figure focusing on the property’s relative production compared to properties of similar age. Hence, the grading scale was not implemented in Sweden until 2014. As the EPC is valid for 10 years, some tenant association–owned buildings only have information for their building’s energy performance, but not the actual grade.

6 Data from Svensk Mäklarstatistik was also used by Wilhelmsson (2019) to study how EPC ratings affect the sales price of single-family houses in Sweden.

7 From January 1, 2019, the way the to measure energy performance in Sweden as displayed by the EPC has changed and is now based on a primary energy number instead of primary energy usage. This will impact the level of energy performance for a small part of our sample with new energy declarations, but the information in the EPC is nevertheless displayed as energy performance in kilowatt-hours per square meter as before. Given that we are using the information from the EPC, we are measuring the signaling effect of energy performance/energy efficiency on the price rather than the actual effect of energy efficiency on the selling price. Hence, this new way to calculate the performance should not be a problem as the energy performance and grades are still displayed in the same way to the homebuyers as before.

8 See Iacus et al. (2012) for more information of how this weight is calculated for the matched observations in the control group.

9 Our cut points are as follows: age (0 14.5 29.5 44.5 59.5 79.5 99.5 120.5), lrent (0 7.7 8 8.3 8.45 8.52 8.7 10), larea (0 3.1 3.45 3.8 4 4.2 4.5 4.75 5 6), and rooms (0 2.25 3.75 5.25 10). For the cut points of the variables with a logarithmic transformation (larea and lrent), these are chosen with respect to corresponding values for the non-logged variables (rent and area). For instance, the range 0–3.1 for larea corresponds to the range 0–22 m^2^ without any logarithmic transformation.
