# Supplementary material for: Does energy efficiency matter for prices of tenant-owned apartments?
Source: Environ Sci Pollut Res Int. 2022 May 4;29(44):66793–807. doi: 10.1007/s11356-022-20482-w (PMC9492582; doi:10.1007/s11356-022-20482-w)
Supplement: Supplementary file 1 — Supplementary file1 (DOCX 29 KB) [file 11356_2022_20482_MOESM1_ESM.docx]

# **Does energy efficiency matter for prices of tenant-owned apartments?**

Appendix

| **Table A1**. Variable summary | |
| --- | --- |
| *Dependent variable* | |
| Price | Sales price (contract price) |
|  |  |
| *Energy efficiency (EPC) variables* | |
| HIGH | Dummy equal to 1 for tenant-owned apartments enclosed in tenant-owned buildings with an energy performance with or below 75 kWh per square meters, else 0. |
|  |  |
| LOW | Dummy equal to 1 for tenant-owned apartments enclosed in tenant-owned buildings with an energy performance over  140 kWh per square meters, else 0. |
|  |  |
| AB | Dummy equal to 1 for tenant-owned apartments enclosed in tenant-owned buildings with energy grade A, B, else equal to 0. |
|  |  |
| ABC | Dummy equal to 1 for tenant-owned apartments enclosed in tenant-owned buildings with energy grade A, B or C, else equal to 0. |
|  |  |
| EFG | Dummy equal to 1 for tenant-owned apartments enclosed in tenant-owned buildings with energy grade E, F or G, else equal to 0. |
|  |  |
| *Dwelling specific control variables* | |
| Area | Area in square meters. |
|  |  |
| Building year | Dummy variables for the building year of the apartments. |
|  |  |
| Rent | Rent or fee paid to the tenant-association each month in SEK. |
|  |  |
| Room | Number of rooms. |
|  |  |
| *Other control variables* | |
| IC 1–3 | Dummy variables for apartments belonging to a DeSO-region with a certain income. We calculate the income quartiles in our sample and create dummy variables based on this. IC1 is equal to 1 for apartments belonging to a postcode with incomes below quartile 1 (<quartile 1 IC 2 is equal to 1 for apartments belonging to a postcode with incomes equal to or larger than quartile 1 but smaller than quartile 2 (income >=quartile 1 & income < quartile 2); IC3 is equal to 1 for apartments belonging to a postcode with incomes equal to or larger than quartile 2 but lower than quartile 3 (income >= quartile 2 & income < quartile 3). |
| Neighborhood controls | The controls includes: median net income (in thousands of SEK), share of persons with foreign origin, share of persons in rental apartments, share of persons receiving financial aid and number of households. In the estimations, we express the net income and number of households in logarithmic form. All the variables that include share of persons is calculated by dividing the total number of persons with that certain characteristic with the total number of persons in the DeSO region. |

| **Table A2.** Summary literature on energy certification and house prices | | | |
| --- | --- | --- | --- |
| ***Authors*** | ***Region and sector*** | ***Country/state*** | ***Conclusion*** |
| Brounen and Kok (2011) | Europe - Residential | Netherlands | Price premiums corresponding to 10, 5 and 2 % respectively for grade A, B, C. |
| Fuerst et al. (2016a) | Europe - Residential | Wales | Premium of 12,8 % for A/B compared to D-rating. |
| De Ayala et al. (2016) | Europe - Residential | Spain | Grade A, B, C or D has a price premium of 5,4 % in comparison with label E, F or G. |
| Hyland et al. (2013) | Europe - Residential | Ireland | 9 % premium for homes with A-label compared to D-level. |
| Fuerst et al. (2016b) | Europe -Residential | Finland (Helsinki) | Price premium of 3,5 % for high efficiency buildings, 1,5 % after neighborhood controls. |
| Fregonara et al. (2017) | Europe - Residential | Italy (Turin) | EPCs does not affects apartment prices. |
| Taltavull et al. (2017) | Europe - Residential | Romania (Bucharest) | Green premium in two out of five investigated areas. |
| Jensen et al. (2016) | Europe - Residential | Denmark | Green premium of 6.2 % (after July 2010) for the grade A/B in comparison with the grade D. |
| Fuerst et al. (2015) | Europe - Residential | England | Price premium for energy efficient buildings in full sample. Uneven effect across property type. |
| Marmolejo-Duarte and Chen (2019) | Europe - Residential | Spain (Barcelona) | Modest effect of energy efficiency. Larger effect for lower-price segment. |
| Bio Intelligence Service (2013) | Europe - Residential | Austria, France, Belgium and UK | Price premium for energy efficient homes in all areas except for Oxford, UK in the range 2.8 - 8 %. |
| Högberg (2013) | Europe - Residential | Sweden (Stockholm) | Small price premium. |
| Cerin et al (2014) | Europe - Residential | Sweden | Premium for energy efficient houses depending on price category and age of building. |
| Wahlström (2016) | Europe - Residential | Sweden | No premium for energy efficient houses. |
| Wilhelmsson (2019) | Europe - Residential | Sweden | Premium of approximately 3 % for energy efficient homes. |
| Kahn and Kok (2014) | US - Residential | California | Small premium. |
| Bruegge et al. (2016) | US - Residential | Florida | New “Energy Star” homes are sold with a premium. |
| Bloom et al. (2011) | US - Residential | Colorado | Premium for Energy Star homes compared to non-certified homes. |
| Australian Bureau of Statistics (2008) | Oceania - Residential | Australia (ACT) | Small premium for green houses. |
| Addae-Dapaah and Chieh (2011) | Asia - Residential | Singapore | Premium for certified homes compared to non-certified. |
| Deng et al. (2012) | Asia - Residential | Singapore | Green premium for certified homes. |
| Zheng et.al (2012) | Asia - Residential | China (Bejing) | Price premium in presale, discount in the resale market. |
| Notes: This literature review is a summary of relevant articles related to our study. We do not claim this to be exhaustive. | | | |

| **Table A3**. Standardized differences in mean (%-bias) between for covariates in matched and unmatched sample | | | | |
| --- | --- | --- | --- | --- |
|  |  |  |  |  |
|  | **Full sample** | | **Graded sample** | |
| Variable | Unmatched | Matched | Unmatched | Matched |
| Room | 32.2 | -4.0 | 17.6 | 5.3 |
| log (Area) | 36.0 | -6.6 | 25.0 | 4.9 |
| log (Rent) | 31.0 | -8.0 | 19.3 | 0.2 |
| Age: |  |  |  |  |
| 1901–1921 | -24.6 | 0.3 | -16.5 | -1.9 |
| 1922–1941 | -48.8 | 0 | -35.4 | -10.6 |
| 1942–1961 | -54.3 | -3.2 | -61.3 | 1.4 |
| 1962–1976 | -26.7 | 3.6 | -5.6 | 2.1 |
| 1977–1991 | -13.3 | -0.4 | -26.0 | 2.9 |
| 1992–2005 | -4.2 | -3.5 | -14.7 | -3.5 |
| 2006– | 144.6 | 3.1 | 103.4 | 5.7 |
| Mean bias | 34.7 | 3.7 | 29.2 | 4.8 |
| Notes: This table shows the standardized differences in mean (% bias) between the control and treatment group in both unmatched and matched samples. Log is the natural logarithm. | | | | |

| **Table A4.** Robustness statistics for CEM | | | | |
| --- | --- | --- | --- | --- |
|  | **Full sample** | | **Graded sample** | |
|  | Unmatched | Matched | Unmatched | Matched |
| Multivariate L1 distance | 0.927 | 0.758 | 0.835 | 0.571 |
|  |  | |  | |
|  | Univariate L1 | | Univariate L1 | |
| Age | 0.58 | 0.34 | 0.55 | 0.09 |
| lrent | 0.13 | 0.10 | 0.14 | 0.09 |
| larea | 0.16 | 0.07 | 0.14 | 0.09 |
| Room | 0.12 | 0.02 | 0.10 | 0.02 |
| Notes: Multivariate L1 represents imbalance with regard to full joint distribution (for how L1 is calculated, see Blackwell et al., 2009). Lower L1 indicates less imbalances in the sample. The univariate L1 represents imbalance regarding the univariate distribution of each covariate. Hence, smaller L1 means less imbalance. | | | | |
